# Gene targeting in adult organs using in vivo cleavable donor plasmids for CRISPR-Cas9 and CRISPR-Cas12a

**DOI:** 10.1038/s41598-024-57551-8

**Published:** 2024-03-31

**Authors:** Riki Ishibashi, Ritsuko Maki, Fumiko Toyoshima

**Affiliations:** 1 Department of Biosystems Science, Institute for Life and Medical Sciences, Kyoto University, Sakyo-ku, Kyoto, 606-8507 Japan; 2 Department of Mammalian Regulatory Networks, Graduate School of Biostudies, Kyoto University, Sakyo-ku, Kyoto, 606-8502 Japan; 3 Department of Homeostatic Medicine, Medical Research Institute, Tokyo Medical and Dental University (TMDU), Yushima, Bunkyo-ku, Tokyo, 113-8510 Japan

**Keywords:** Gene delivery, Genetic engineering, Genetic techniques, Animal biotechnology, Biomaterials, Gene therapy, Genomics

## Abstract

The CRISPR-Cas system for in vivo genome editing is a powerful tool for gene therapy against several diseases. We have previously developed the pCriMGET_9-12a system, an in vivo cleavable donor plasmid for precise targeted knock-in of exogenous DNA by both Cas9 and Cas12a. Here, we show that the pCriMGET_9-12a system can be applied for in vivo in-frame knock-in of exogenous DNA in adult mouse liver by hydrodynamic delivery of the targeting plasmids. The in vivo cleavable pCriMGET_9-12a donor plasmids significantly increased the knock-in efficiency of both CRISPR-Cas9 and CRISPR-Cas12a in the adult mouse liver compared to uncleavable donor plasmids. This strategy also achieved in-frame reporter gene knock-in without indel mutations. Therefore, in vivo gene targeting using the pCriMGET_9-12a system may contribute to the establishment of safer, more precise, versatile and efficient gene therapy methods in adult organs.

## Introduction

Clustered regularly interspaced short palindromic repeats (CRISPR)-CRISPR-associated (Cas) systems-mediated in vivo genome editing strategies are expected to treat several genetic diseases^1–4^. In particular, homology directed repair (HDR) can repair small or large deletions or induce stable therapeutic gene expression^5^. On the other hand, low knock-in efficiencies in regions inaccessible to CIRSPR-Cas9 due to protospacer adjacent motif (PAM) sequence restriction and incorrect and random integration due to non-homologous end joining of the donor cassette are issues in gene therapy^6–9^.

CRISPR-Cas12a recognises T-rich PAM sequences differently from CRISPR-Cas9 and is therefore used as a tool to enable genome editing in genomic regions that cannot be cleaved by CRISPR-Cas9^10–12^. In addition, CIRSPR-Cas12a has been reported to have fewer off-target effects on the human genome than CRISPR-Cas9^13,14^, and its smaller nuclease size is thought to be advantageous over CIRSPR-Cas9 for vector construction and in vivo delivery^15^. The CRISPR-Cas12a system could improve the low knock-in efficiency of CRISPR-Cas9 at uncleavable gene loci, but the insertion of large DNA fragments by CRISPR-Cas12a in living organs has not been demonstrated.

Hydrodynamic delivery is an effective and non-viral method of naked DNA transfer to the liver in live animals^16,17^. In this method, a physical force derived from the rapid injection of a large volume of DNA solution equivalent to 8–10% of body weight into the vein within 5–10 s induces effective gene delivery and expression. Hydrodynamic tail vein injection (HTVi) of plasmid DNA is typically and widely used to induce gene expression in the liver of small rodents^18–20^. In addition, hydrodynamic retro-orbital sinus injection has been reported to deliver naked plasmid DNA to the liver at levels comparable to HTVi^21^. Furthermore, hydrodynamic delivery has been extended beyond naked DNA to include RNA^22–27^, small molecules^28,29^, proteins^28,30^, viruses^31^ and cells^32^. This non-viral delivery strategy is expected to be safer than viral-based delivery methods for human gene therapy.

Previously, we developed an in vivo cleavable donor plasmid, pCriMGET_9-12a (plasmid of synthetic CRISPR-coated RNA target sequence-equipped donor plasmid-mediated gene targeting via Cas9 and Cas12a), for CRISPR-Cas9 and Cas12a genome editing^33^. The pCriMGET_9-12a system allowed precise knock-in of long (4.0–5.4 kb) exogenous DNA by both CRISPR-Cas9 and CRISPR-Cas12a in cultured cells and mouse zygotes. Here, we combined the pCriMGET_9-12a system with a hydrodynamic-based naked plasmid DNA delivery method and succeeded in precise knock-in of reporter genes into the targeted genomic locus by both CRISPR-Cas9 and CRISPR-Cas12a in the liver of adult mice.

## Results

### Hydrodynamic-based plasmid DNA delivery via retro-orbital sinus injection

**Figure 1:**
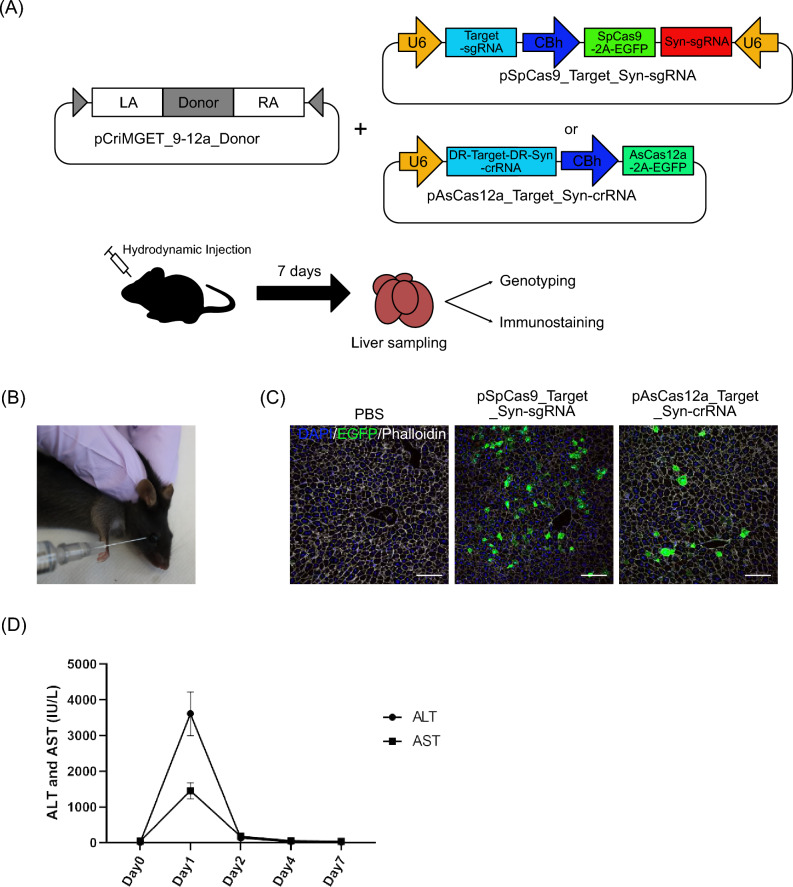
Hydrodynamic based retro-orbital sinus injection with the pCriMGET_9-12a system. (**A**) Schematic of the combination with hydrodynamic retro-obital sinus injection and the pCriMGET_9-12a system. pCriMGET_9-12a_donor and pSpCas9_Target_Syn-sgRNA, expressed as SpCas9-2A-EGFP and target-sgRNA and Syn-crRNA-TS-sgRNA, or pAsCas12a_Target_Syn-crRNA, expressed as AsCas12a-2A-EGFP and target-crRNA and Syn-crRNA-TS-crRNA, are injected from the retro-orbital sinus. The liver was corrected for genotyping and immunostaining on day 7 after injection. (**B**) Hydrodynamic retro-orbital sinus injection technique. (**C**) Representative immunofluorescence images of mouse liver sections injected with PBS, pSpCas9_Target_syn-sgRNA or pAsCas12a_Target_Syn-crRNA on one day after injection. EGFP (green), phalloidin (white) and DAPI (blue). Scale bar, 20 μm. The experiment was repeated in 4 individual mice. (**D**) Serum levels of ALT and AST after retro-orbital sinus injection of plasmid DNA. Day 0 as uninjected control. The experiment was repeated in 3 individual mice at each time point.

For gene targeting with the pCriMGET_9-12a system in the liver of adult mice, we evaluated the delivery efficiency of naked plasmid DNA by hydrodynamic injection methods. According to the previous report, the plasmid delivery efficiency of hydrodynamic injection via the retro-orbital sinus is comparable to that of traditional HTVi method^21^. We constructed pCriMGET_9-12a donor carrying the 500-bp homology arms, and pSpCas9_Target_Syn-sgRNA and pAsCas12a_Target_syn-cRNA plasmids, which encode the *SpCas9-2A-EGFP* or *AsCas12a-2A-EGFP* gene downstream of the chicken β-actin hybrid (CBh) promoter together with the Target_Syn-sgRNA or Target_Syn-crRNA sequence downstream of the U6 promoter (Fig. 1A). The plasmids were then administrated to the adult mice by retro-orbital sinus hydrodynamic injection (Fig. 1B). EGFP expression, an indicator of SpCas9 or AsCas12a expression, was observed in liver hepatocytes 24 h after injection, which shows that both plasmids were delivered to the liver and Cas9 or Cas12a protein was expressed in the hepatocytes of adult mice (Fig. 1C). To assess the potential hepatotoxicity of this procedure, serum levels of alanine aminotransferase (ALT) and asparate aminotransferase (AST), biomarkers of liver damage and inflammation, were measured. Both ALT and AST levels increased one day after injection, followed by a return to baseline levels by day 4 (Fig. 1D). This suggests that, as previously reported^21^, hydrodynamic retro-orbital sinus injection induces acute liver damage or inflammation, but the effect is transient.

### Precise in-frame knock-in of the reporter cassette for tagging endogenous gene using the pCriMGET_9-12a/CRISPR-Cas9 system

**Figure 2:**
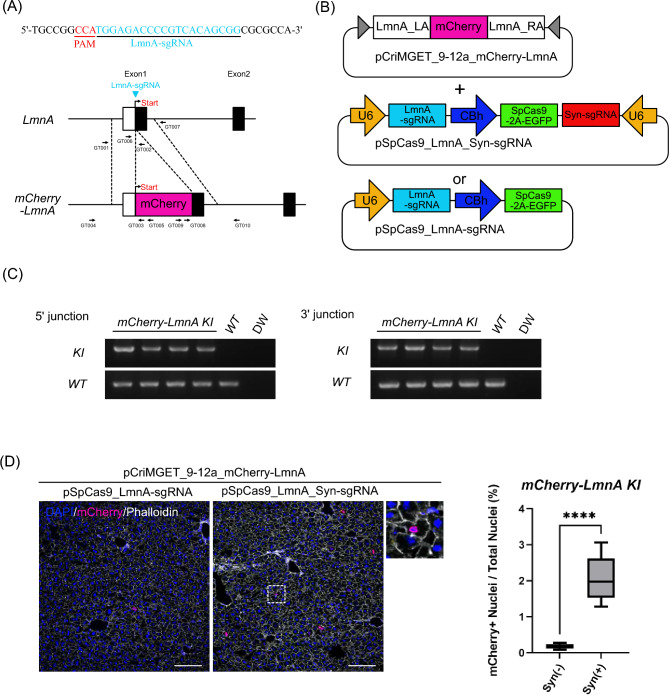
Precise in-frame knock-in of *mCherry-LmnA* using the pCriMGET_9-12a/CRISPR-Cas9 systems. (**A**) Targeting map of *mCherry-LmnA*. The LmnA sgRNA target site (blue arrow head) and sequence (blue letters), and the CRISPR-Cas9 PAM sequence (red letters) are shown. (**B**) Use of plasmids for *mCherry-LmnA* gene targeting. (**C**) Genotyping PCR for *mCherry-LmnA* knock-in liver. PCR for WT band detection was performed using LmnA-mCherry_GT001 and _GT002 primers for 5′ junction loci and LmnA-mCherry_GT006 and _GT007 primers for 3′ junction loci. PCR for KI band detection was performed using LmnA-mCherry_GT004 and _GT005 primers for 1st PCR and _GT001 and _GT003 primers for nested PCR at 5′ junction loci, and LmnA-mCherry_GT009 and _GT0010 primers for 1st PCR and _GT007 and _GT008 primers for nested PCR at 3′ junction loci (see (**A**)). (**D**) Representative immunofluorescence images of liver tissue injected with pCriMGET_9-12a_mCherry-LmnA and pSpCas9_LmnA-sgRNA or pSpCas9_LmnA_Syn-sgRNA plasmids. DAPI (blue), mCherry (magenta) and phalloidin (white). Scale bar, 20 μm. Enlarged image is shown as dashed box area. Knock-in ratio was calculated as the percentage of mCherry + nuclei among total nuclei (n > 13,000 nuclei from four individual slides). Mean ± s.d. from four individual slides. ****P < 0.001, by two-tailed Student's t-test.

We investigated whether pCriMGET_9-12a with CRISPR-Cas9 could be used for precise in-frame insertion of a reporter gene tag into an endogenous gene locus in the liver of adult mice. We designed a strategy for targeted knock-in of the *mCherry* reporter gene into the *Lamin A* (*LmnA*) gene locus on the mouse genome (Fig. 2A). We constructed the donor plasmid pCriMGET_9-12a_mCherry-LmnA, which contains the *mCherry* gene upstream of the endogenous *LmnA* coding sequence flanked by 500 bp homology arms. The donor plasmid was delivered into the liver of adult mice via retro-orbital sinus hydrodynamic injection together with pSpCas9_LmnA_Syn-sgRNA or pSpCas9_LmnA-sgRNA (Fig. 2B). The EGFP signal, which indicates SpCas9 expression, was detected in approximately 7% of hepatocytes one day after injection. However, it was barely detectable 7 days after injection (Supplementary Fig. S1, GFP). In contrast, the mCherry signal became detectable 7 days after injection (Supplementary Fig. S1, mCherry). These results suggest that the SpCas9 protein was expressed transiently and that knock-in of the *mCherry* gene was induced 7 days after plasmid injection. To assess the accuracy of the targeted knock-in, the liver was harvested 7 days after plasmid injection and PCR genotyped at the 5′ and 3′ junction loci. The result showed that the PCR amplicon specific for the knock-in was detected in the liver injected with pCriMGET_9-12a_mCherry-LmnA and pSpCas9_LmnA_Syn-sgRNA plasmids (Fig. 2C, Supplementary Fig. S2A,B). In addition, no indels or frameshifts were detected in the 5′ and 3′ junction regions of the knock-in-specific amplicon (Supplementary Fig. S3). Furthermore, mCherry signal was detected on the nuclear envelope in hepatocytes, which recapitulates the localization of endogenous LmnA protein^34^ (Fig. 2D). Importantly, the knock-in frequency was significantly higher in the presence of Syn-sgRNA than in its absence (Fig. 2D). Flow cytometric analysis of whole liver cells showed that the percentage of mCherry-positive cells was again significantly higher in the presence of Syn-sgRNA than in its absence (Supplementary Fig. S4). These results show that the pCriMGET_9-12a/CRISPR-Cas9 system achieves precise knock-in of the reporter gene into the targeted genomic locus in the adult mouse liver.

### Precise integration of the bicistronic reporter gene cassette into the targeted genomic locus using the pCriMGET_9-12a/CRISPR-Cas12a system

**Figure 3:**
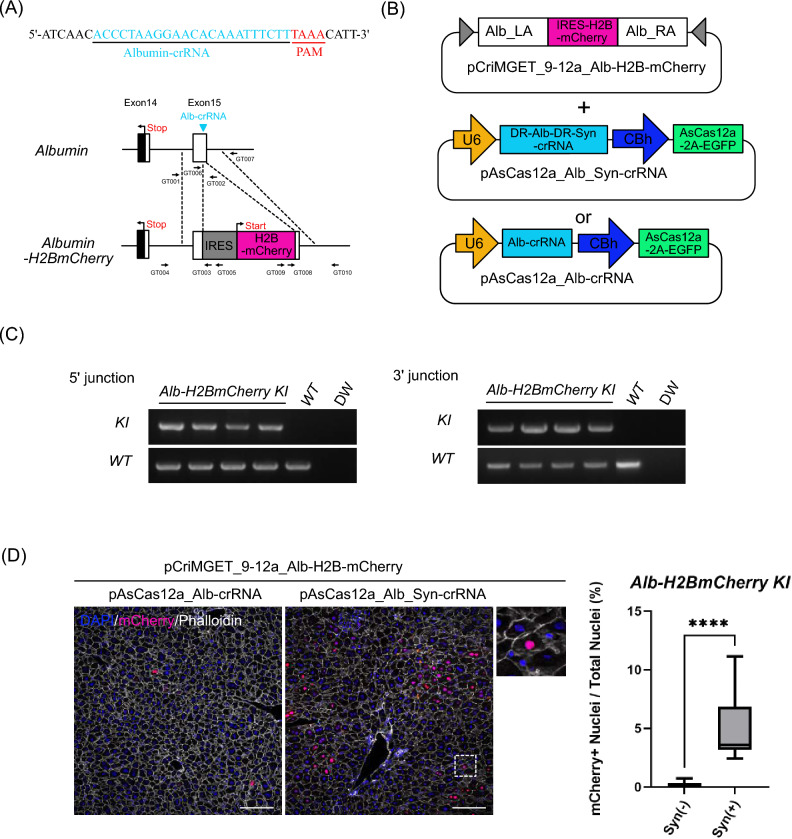
Bicistronic *H2B-mCherry* reporter cassette targeting the albumin 3′ UTR locus by the pCriMGET_9-12a/CRISPR-Cas12a system. (**A**) Targeting map of *Albumin-H2BmCherry*. Alb-crRNA target site (blue arrow head) and sequence (blue letters), and CRISPR-Cas12a PAM sequence (red letters) are shown. (**B**) Use of plasmids for *Albumin-H2BmCherry* gene targeting. (**C**) Genotyping PCR for *Alb-H2BmCherry* knock-in liver. PCR for WT band detection was performed using Alb-H2BmCherry_GT001 and _GT002 primers for 5′ junction loci and Alb-H2BmCherry _GT006 and _GT007 primers for 3′ junction loci. PCR for KI band detection was performed using Alb-H2BmCherry_GT004 and _GT005 primers for 1st PCR and _GT001 and _GT003 primers for nested PCR at 5′ junction loci, and Alb-H2BmCherry _GT009 and _GT0010 primers for 1st PCR and _GT007 and _GT008 primers for nested PCR at 3′ junction loci (see (**A**)). (**D**) Representative immunofluorescence images of liver tissue injected with pCriMGET_9-12a_ Alb-H2BmCherry and pAsCas12a_Alb-crRNA or pAsCas12a_Alb_Syn-crRNA plasmids. DAPI (blue), mCherry (magenta) and phalloidin (white). Scale bar, 20 μm. Enlarged image is shown as dashed box area. Knock-in ratio was calculated as the percentage of mCherry + nuclei among total nuclei (n > 12,000 nuclei from four individual slides). Mean ± s.d. from four individual slides. ****P < 0.001, by two-tailed Student's t-test.

We then investigated whether pCriMGET_9-12a with CIRSPR-Cas12a could also be used to integrate an exogenous DNA cassette into an endogenous genomic locus in the liver of adult mice. We designed a strategy for targeted knock-in of the bicistronic reporter gene cassette, which induces H2B-mCherry expression via the inter-ribosomal entry site (IRES)^35^, into the *albumin* (*Alb*) gene locus on the mouse genome (Fig. 3A). We constructed the donor plasmid pCriMGET_9-12a_Alb-H2B-mCherry, which integrates the *IRES-H2B-mCherry* gene into the endogenous *albumin* 3′ UTR loci flanked by 500 bp homology arms (Fig. 3B). The donor plasmid was delivered into the liver of adult mice by retro-orbital sinus hydrodynamic injection together with pAsCas12a_Alb_Syn-crRNA or pAsCas12a_Alb-crRNA (Fig. 3B). The liver was harvested 7 days after plasmid injection and PCR genotyped at the 5′ and 3′ junction loci. The results showed that the PCR amplicon specific for the knock-in was detected in liver injected with pCriMGET_9-12a_Alb-H2B-mCherry and pAsCas12a_Alb_Syn-crRNA plasmids (Fig. 3C, Supplementary Fig. S2C,D). Furthermore, no indels or frameshifts were detected in the 5′ and 3′ junction regions of the knock-in-specific amplicon (Supplementary Fig. S5). In addition, H2B-mCherry signal was detected in the nucleus of hepatocytes, and the knock-in frequency was significantly higher in the presence of Syn-crRNA than in its absence (Fig. 3D). Flow cytometric analysis of whole liver cells showed that the percentage of mCherry-positive cells was again significantly higher in the presence of Syn-crRNA than in its absence (Supplementary Fig. S6). These results show that the pCriMGET_9-12a/CRISPR-Cas12a system can be used for the integration of bicistronic reporter gene cassettes into the targeted genomic locus in the adult mouse liver.

### The pCriMGET_9-12a/CRISPR-Cas12a system enables precise in-frame knock-in of reporter cassette for tagging endogenous gene

**Figure 4:**
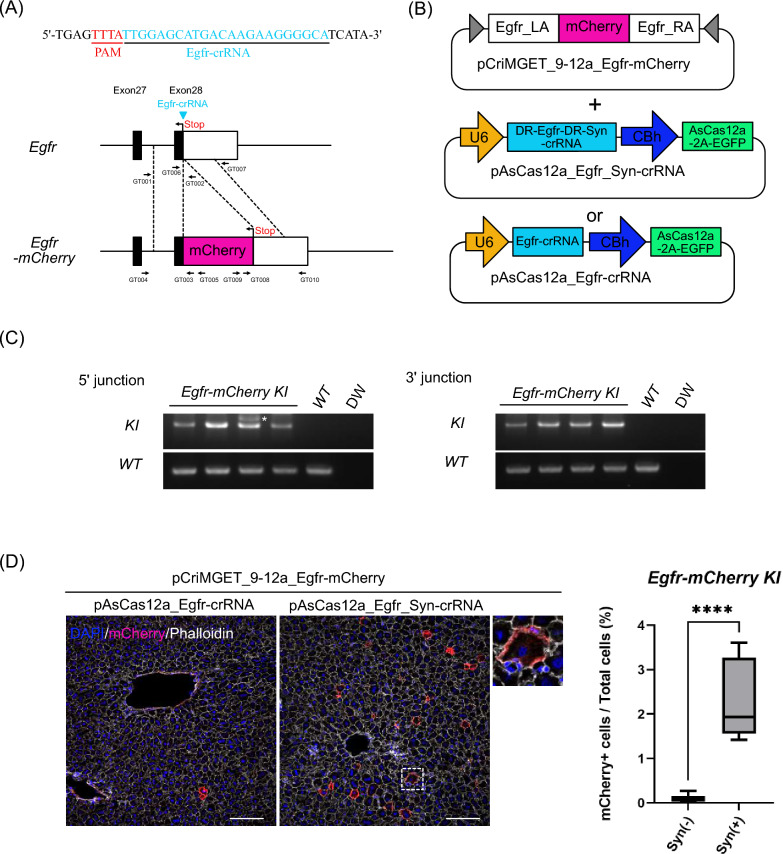
Precise in-frame knock-in of *Egfr-mCherry* by the pCriMGET_9-12a/CRISPR-Cas12a systems. (**A**) Targeting map of *Egfr-mCherry*. Egfr-crRNA target site (arrowhead) and sequence (blue letters), and CRISPR-Cas12a PAM sequence (red letters) are shown. (**B**) Use of plasmids for *Egfr-mCherry* gene targeting. (**C**) Genotyping PCR for *Egfr-mCherry* knock-in liver. PCR for WT band detection was performed using Egfr-mCherry _GT001 and _GT002 primers for 5′ junction loci and Egfr-mCherry _GT006 and _GT007 primers for 3′ junction loci. PCR for KI band detection was performed using Egfr-mCherry _GT004 and _GT005 primers for 1st PCR and _GT001 and _GT003 primers for nested PCR at 5′ junction loci, and Egfr-mCherry _GT009 and _GT0010 primers for 1st PCR and _GT007 and _GT008 primers for nested PCR at 3′ junction loci (see (**A**)) Asterisk indicates non-specific amplicon. (**D**) Representative immunofluorescence images of liver tissue injected with pCriMGET_9-12a_Egfr-mCherry and pAsCas12a_Egfr-crRNA or pAsCas12a_Egfr_Syn-crRNA plasmids. DAPI (blue), mCherry (magenta) and phalloidin (white). Scale bar, 20 μm. Enlarged image is shown as dashed box area. Knock-in ratio was calculated as the percentage of mCherry + cells among total cells (n > 13,000 cells from four individual slides). Mean ± s.d. from four individual slides. ****P < 0.001, by two-tailed Student's t-test.

Finally, we examined whether pCriMGET_9-12a with CIRSPR-Cas12a could achieve in-frame knock-in for tagging the endogenous gene with the exogenous reporter cassette in adult mouse liver. We designed a strategy for targeted knock-in of the mCherry reporter gene cassette into the *Epidermal growth factor receptor* (*Egfr*) gene locus on the mouse genome (Fig. 4A). We constructed the donor plasmid pCriMGET_9-12a_Egfr-mCherry, which contains the *mCherry* gene downstream of the endogenous *Egfr* coding sequence flanked by 500 bp homology arms (Fig. 4B). The donor plasmid was delivered into the liver of adult mice by retro-orbital sinus hydrodynamic injection together with pAsCas12a_Egfr_Syn-crRNA or pAsCas12a_Egfr-crRNA (Fig. 4B). The liver was harvested 7 days after plasmid injection and PCR genotyped at the 5′ and 3′ junction loci. The results showed that the PCR amplicon specific for the knock-in was detected in the liver injected with pCriMGET_9-12a_Egfr-mCherry and pAsCas12a_Egfr_Syn-crRNA plasmids (Fig. 4C, Supplementary Fig. S2E,F). Furthermore, no indels or frameshifts were detected in the 5′ and 3′ junction regions of the knock-in-specific amplicon (Supplementary Fig. S7). In addition, mCherry signal was detected at the plasma membrane in hepatocytes, which recapitulates the localization of endogenous Egfr protein^36^. Importantly, the knock-in frequency in the presence of Syn-crRNA was significantly higher than in its absence (Fig. 4D). Flow cytometric analysis of whole liver cells showed that the percentage of mCherry-positive cells was again significantly higher in the presence of Syn-crRNA than in its absence (Supplementary Fig. S8). These results show that the pCriMGET_9-12a/CRISPR-Cas12a system also achieves in-frame knock-in of the reporter gene cassettes into the targeted genomic locus in the adult mouse liver.

## Discussion

The CRISPR-Cas-mediated in vivo genome editing of adult organs has been developed by using virus-based delivery techniques^37,38^. Although adeno-associated virus (AAV) vectors and adenovirus (AdV) vector gene delivery systems have achieved highly efficient CRISPR-Cas component delivery in vivo^39–42^, several problems have been reported in the context of gene therapies; (1) the host immune system produces neutralising antibodies against viral particles, reducing the efficiency of viral delivery into target cells with repeated administration^42–47^, (2) the administration of a high dose of the AAV vector induces hepatotoxicity^48^, (3) sustained expression of the CIRPSR-Cas nuclease from the viral vector in host cells has been concerned to induce unwanted mutations and random integration of viral genomic fragments into the host genome^49–51^. In this study, we proposed the versatile gene targeting methods by delivering naked plasmid DNA into adult organs. The previous study showed that the homology-mediated end joining (HMEJ) method via CRISPR-Cas9 by HTVi achieved effective and precise reporter gene knock-in in adult mouse liver^52^. The knock-in efficiency and accuracy of the pCriMGET_9-12a system is comparable to the HMEJ method, with CRISPR-Cas12a as well as CIRSPR-Cas9. Although it has been reported that CRISPR-Cas12a has succeeded in effective gene disruption in vivo^53–55^, there are no reports of achieving CRISPR-Cas12a-mediated exogenous DNA knock-in in vivo. As far as we know, this study is the first report of CRISPR-Cas12a-mediated in-frame knock-in of a 0.7–1.7 kb double-stranded DNA cassette in the liver of adult mice.

Limitations of the method developed in this study include the low knock-in efficiency and the limited number of target organs. Plasmid DNA delivery was achieved in only about 7% of the hepatocytes (Supplementary Fig. S1), suggesting that the low knock-in efficiency may be due to limited plasmid DNA delivery. Furthermore, the previous study shows that plasmid DNA delivered by hydrodynamic injection accumulates not only in hepatocytes but also in non-parenchymal cells^56^, although the majority of the knock-in cells in our study appear to be hepatocytes based on the results of immunostaining and flow cytometry (Figs. 2D, 3D, 4D, Supplementary Figs. S4, S6, S8). By using nanoparticles, a recently developed delivery vehicle for non-viral DNA carriers for specific organs and cells^57–60^, the pCriMGET_9-12a system would be expected to be an effective gene targeting system in multiple organs and cells. It is also expected to be used to deliver plasmid DNA in a safer way compared to hydrodynamic injection. Another limitation is the possibility of random integration, as suggested by non-specific amplicon in PCR genotyping analysis (Supplementary Fig. S2). For the application of this system in gene therapy, it is necessary to reduce random integrations and to establish the detection method of these mutations. Our study may contribute to expand the editable gene loci and repair large deleted genome, and open new avenues for developing safer non-viral gene therapy method.

## Methods

### pCriMGET_9-12a_mCherry-LmnA

mCherry was amplified by PCR using pCriMGET_9-12a_Rosa26-CAG-LSL-NuM-mCherry-WPRE-pA^33^ as a template. The 3xGS linker-coading sequence (5′-GGCAGCGGCAGCGGCAGC-3′) was fused to the 3′ end of the mCherry gene by PCR amplification. The mouse genomic DNA sequences 500 bp upstream and downstream of the sgRNA target site of the LmnA gene locus were amplified by PCR using C57BL/6JmsSlc mouse genomic DNA as a template and were used as the left and right homology arms, respectively. The fragments were fused and inserted into the *EcoRV* site of the pCriMGET_9-12a multiple cloning site using NEBuilder HiFi DNA Assembly Master Mix (New England Biolabs) according to the manufacturer's protocol.

### pCriMGET_9-12a_Alb-H2B-mCherry

IRES sequence was amplified by PCR using the G13 sensor (Ga13_2) (Addgene #112930)^61^ as a template. H2B-mCherry was amplified by PCR using the pCriMGET_9-12a_Rosa26-CAG-LSL-NuM-mCherry-WPRE-pA template^33^. The mouse genomic DNA sequences 500 bp upstream and downstream of the crRNA target site of the albumin gene locus were amplified by PCR using C57BL/6JmsSlc mouse genomic DNA as a template and were used as the left and right homology arms, respectively. The fragments were fused and inserted into the *EcoRV* site of the pCriMGET_9-12a multiple cloning site using NEBuilder HiFi DNA Assembly Master Mix (New England Biolabs) according to the manufacturer's protocol.

### pCriMGET_9-12a_Egfr-mCherry

mCherry was amplified by PCR using the pCriMGET_9-12a_Rosa26-CAG-LSL-NuM-mCherry-WPRE-pA template ^33^. The 3xGS linker-coading sequence (5′-GGCAGCGGCAGCGGCAGC-3′) was fused to the 5′ end of the mCherry gene by PCR amplification. The mouse genomic DNA sequences 500 bp upstream and downstream of the sgRNA target site of the Egfr gene locus were amplified by PCR using C57BL/6JmsSlc mouse genomic DNA as a template and were used as the left and right homology arms, respectively. The fragments were fused and inserted into the *EcoRV* site of the pCriMGET_9-12a multiple cloning site using NEBuilder HiFi DNA Assembly Master Mix (New England Biolabs) according to the manufacturer's protocol.

### pSpCas9_LmnA-sgRNA, and pSpCas9_LmnA-Syn-sgRNA

The pSpCas9_target-Syn-sgRNA plasmid was constructed as follows: The U6 promoter-syn-sgRNA sequence was amplified by PCR using pX330.1-syn-crRNA-TS-sgRNA^33^ as a template. The fragment was then inserted into the *NotI* site of pX330.1^33^ using NEBuilder HiFi DNA Assembly Master Mix (New England Biolabs). The oligonucleotides LmnA-sgRNA (5′-CCGCTGTGACGGGGTCTCCA-3′) were annealed and ligated into the *BbsI* site of pX330.1 and pSpCas9_target-Syn-sgRNA plasmids, respectively.

### pAsCas12a_Alb-crRNA, pAsCas12a_Alb_Syn-crRNA, pAsCas12a_Egfr-crRNA, and pAsCas12a_Egfr_Syn-crRNA

The oligonucleotides Alb-crRNA (5′-AAGAAATTTGTGTTCCTTAGGGT-3′), Alb_Direct repeat (DR)_Syn-crRNA (5′-AAGAAATTTGTGTTCCTTAGGGTTAATTTCTACTCTTGTAGATGCTGTCCCCAGTGCATATTCAGG-3′) and Egfr-crRNA (5′-TTGGAGCATGACAAGAAGGGGCA-3′), Egfr_Direct Repeat (DR)_Syn-crRNA (5′-TTGGAGCATGACAAGAAGGGGCATAATTTCTACTCTTGTAGATGCTGTCCCCAGTGCATATTCAGG-3′) were annealed and ligated into the *BsmBI* site of pY094. 1^33^, respectively.

### Retro-orbital sinus hydrodynamic injection

10 µg of pCriMGET_9-12a donor plasmids and 20 µg of pSpCas9 or pAsCas12a plasmids were diluted with 2 mL of PBS(-) (Nacalai Tesque) at room temperature. 6-week-old mice were anaesthetised with 1.5% isoflurane, and then the plasmid DNA was rapidly injected into the retro-orbital sinus using a 27-gauge needle under the restraint of a researcher's hand.

### Assessment of liver injury and inflammation

Mouse serum was prepared by collecting coagulated whole blood from uninjected control mice and the mice injected with plasmid DNA at 1, 2, 4 and 7 days after injection. Serum levels of AST and ALT were determined by Oriental yeast co., LTD.

### Animals used in this study

C57BL/6JJmsSLC mice were obtained from Japan SLC Inc. (Shizuoka, Japan). All of the experiments were performed in accordance with ARRIVE guidelines and the guidelines of the Kyoto University Regulation on Animal Experimentation, and were approved by the Committee for Animal Experiments of the Institute for Life and Medical Sciences, Kyoto University (A21-2-2).

### Genotyping PCR

Genomic DNA was extracted from mouse liver by the classical organic extraction method using phenol/chloroform/isoamyl alcohol. A piece of liver was homogenised and digested overnight at 55℃ with 500μL lysis buffer (20 mM Tris–Cl (pH8.0), 5 mM EDTA (pH8.0), 400 mM NaCl, 0.3% SDS) containing 200 μg/mL protenase K (Nacalai Tesque). The lysate was purified with 500μL phenol/chloroform/isoamyl alcohol 25:24:1 (Nacalai Tesque) and concentrated by ethanol precipitation. PCR was performed with 20 ng genomic DNA using KOD One Blue PCR Master Mix (TOYOBO). To detect the WT amplicon, PCR was performed as follows; GT001 and GT002 primers were used to amplify the 5′ junction loci and GT006 and GT007 primers were used to amplify the 3′ junction loci in each sample. To detect knock-in amplicons, nested PCR was performed as follows: GT004 and GT005 primers were used to amplify 5′ junction loci in each knock-in in the first PCR. GT009 and GT010 primers were used to amplify the 3′ junction loci in each knock-in in the first PCR. After the first PCR amplification, they were diluted 1:100 in DW as a nested PCR template. GT001 and GT003 primers were then used to amplify the 5′ junction loci in each knock-in in a nested PCR. GT007 and GT008 primers were used to amplify 3′ junction loci in each knock-in in a nested PCR. The list of primers for genotyping is shown in Supplementary Table S1.

### Sequence analysis

Knock-in-specific PCR amplicons were purified using the QIAquick Gel Extraction Kit (QIAGEN) according to the manufacturer's protocol. Sanger sequencing was performed by Azenta Life Sciences. All data analysis was performed using FinchTV software. The list of primers for Sanger sequencing is shown in Supplementary Table S1.

### Immunohistochemistry

After 1 day and 7 days of injection, Optimal cutting temperature compound was used to embed and freeze the livers of mice injected with plasmid DNA. The samples were sectioned, immunostained, fixed with 4% paraformaldehyde and permeabilized with 0.5% Triton X-100 in Tris-buffered saline for 15 min at room temperature. Sections were then blocked with Blocking-One Histo (Nacalai Tesque) for 1 h at room temperature, incubated with primary antibodies overnight at 4 °C, washed and incubated with secondary antibodies for 1 h. The samples were mounted using Fluoromount-G™ Mounting Medium with DAPI (Invitrogen). Primary antibodies were anti-EGFP (chicken, 1:1000, ab13970; Abcam), anti-mCherry (rabbit, 1:500, 26765-1-AP; Proteintech^®^). Secondary antibodies were Alexa Fluor 488-conjugated anti-chicken, Cy3-conjugated anti-rabbit (Jackson ImmunoResearch, West Grove, PA, USA). F-actin was labeled with Alexa Fluor 647-conjugated Phalloidin (A22287; Invitrogen). Each primary and secondary antibody was diluted with Can Get Signal® Immunostain Immunoreaction Enhancer Solution B (TOYOBO). All images were captured using an Olympus FV3000 confocal microscope and subjected to knock-in efficiency quantification. Four random images were taken from mouse liver sections and the total number of nuclei in each image was counted. In addition, knock-in efficiency was calculated by dividing the number of cells expressing the reporter gene by the number of all nuclei counted, using the Cy3 channel signal as an indicator. The total number of nuclei and cells expressing the reporter gene were counted using Olympus cellSense Count and Measure software. All experiments were repeated at least four times in independent mice.

### Flow cytometric analysis

Whole hepatocytes were obtained from mouse liver using a collagenase digestion method. Briefly, mice were laparotomised under isoflurane inhalation anaesthesia, and the portal vein was cannulated and perfused with PBS. Liver tissues were then cut into small pieces and transferred to liver digestion medium (D-MEM (Nacalai) with 10% fetal bovine serum (Sigma) and 1 mg/mL collagenase (WAKO)) and incubated at 37℃ with continuous shaking for 60 min. The samples were clarified using a cell strainer (100 μm) (BD). After washing with phosphate-buffered saline containing 1% bovine serum albumin, cells were analysed using BD LSR-Fortessa X-20 (BD). Dead cells were stained with SYTOX^®^ Blue Dead Cell Stain (Invitrogen).

### Statics and reproducibility

All animal experiments were performed independently at least four times on different mice. All experiments were successful. The sample size and statistical analysis used for each quantification are indicated in the figure legends. No animals or data were excluded from the analysis. Images and samples were randomly selected and analysed equally. Data collection and analysis for genotyping, imaging and quantification were not blinded to experimental conditions. Reproducibility was confirmed by two investigators in independent experiments. All statistical analyses were performed with GraphPad Prism 8 or Microsoft Excel.

### Supplementary Information

Supplementary Information.

## Data Availability

All data generated or analyzed during this study are included in this published article and available from the corresponding author on reasonable request.
