# Supplementary material for: Gene targeting in adult organs using in vivo cleavable donor plasmids for CRISPR-Cas9 and CRISPR-Cas12a
Source: Sci Rep. 2024 Mar 31;14:7615. doi: 10.1038/s41598-024-57551-8 (PMC10982285; doi:10.1038/s41598-024-57551-8)
Supplement: Supplementary file 1 — Supplementary Information. [file 41598_2024_57551_MOESM1_ESM.pdf]

Supplementary information

Gene targeting in adult organs in vivo cleavable donor plasmids for CRISPR-Cas9 and CRISPR-Cas12a.

Riki Ishibashi[1,2]*, Ritsuko Maki[1], and Fumiko Toyoshima[1,2,3]

[1]Department of Biosystems Science, Institute for Life and Medical Sciences, Kyoto University, Sakyo-ku, Kyoto 606-8507, Japan

[2]Department of Mammalian Regulatory Networks, Graduate School of Biostudies, Kyoto University, Sakyo-ku, Kyoto 606-8502, Japan

[3]Department of Homeostatic Medicine, Medical Research Institute, Tokyo Medical and Dental University (TMDU), Yushima Bunkyo-ku Tokyo 113-8510 Japan

*Corresponding author:
Riki Ishibashi
E-mail: rishibas@infront.kyoto-u.ac.jp
Tel.: +81-75-751-4016
Fax: +81-75-751-4037

This PDF file includes :

Supplementary Figure S1 to S8

Supplementary Table S1

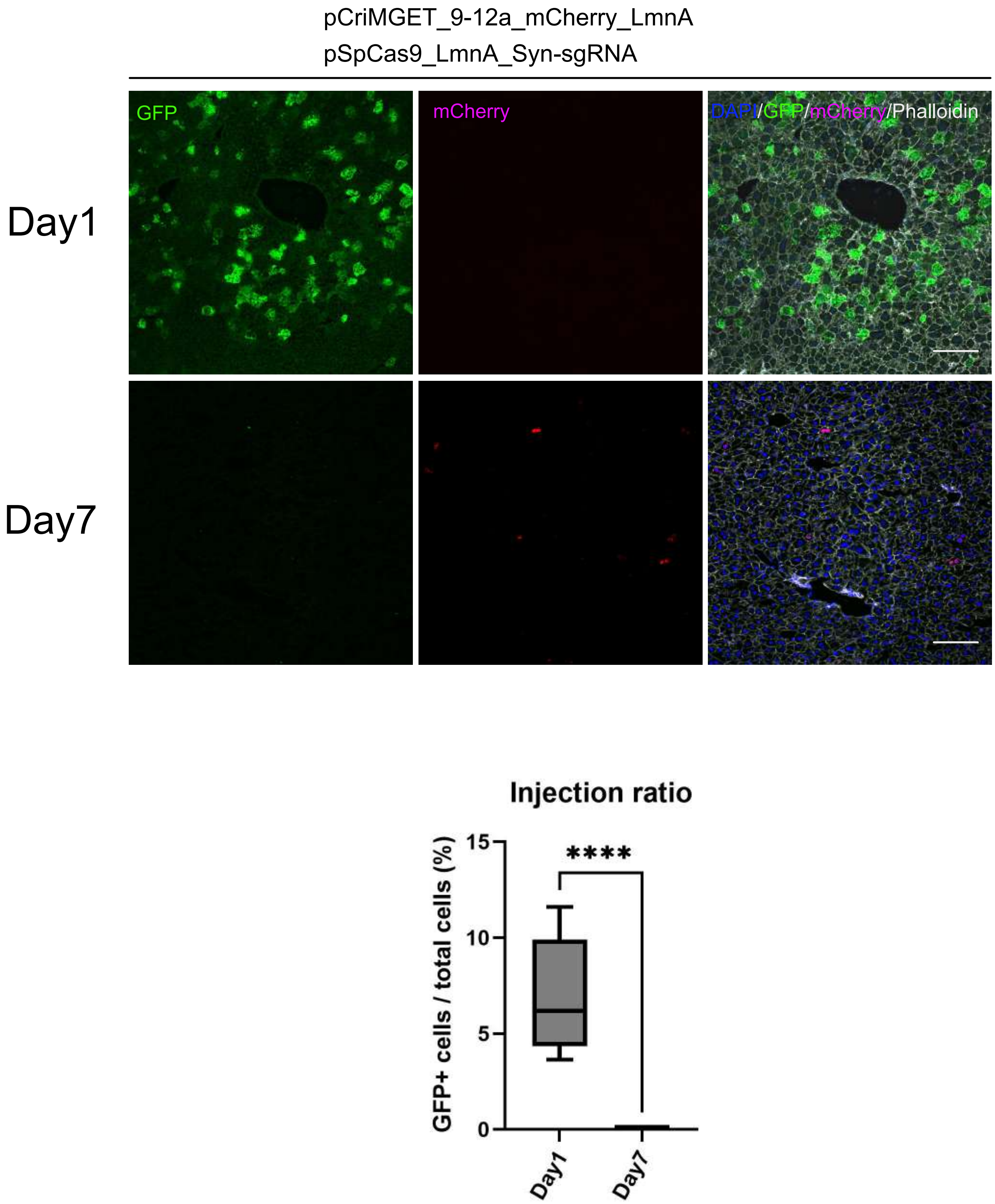

Supplementary Figure S1. pCriMGET_9-12a_mCherry_LmnA and pSpCas9_LmnA _Syn-sgRNA plasmids delivery into liver via hydrodynamic-based retro-orbital sinus injection. (A) Representative immunofluorescence images of liver tissue injected with pCriMGET_9-12a_mCherry-LmnA and pSpCas9_ LmnA_Syn-sgRNA plasmids at day1 and Day7 after injection. DAPI (blue), GFP (green), mCherry (magenta) and phalloidin (white). Scale bar, 20μm. Injection ratio was calculated as the percentage of GFP+ cells among total cells (n > 13,000 cells from four individual slides). Mean ± s.d. from four individual slides. ****P <0.001, by two-tailed Student's t-test.

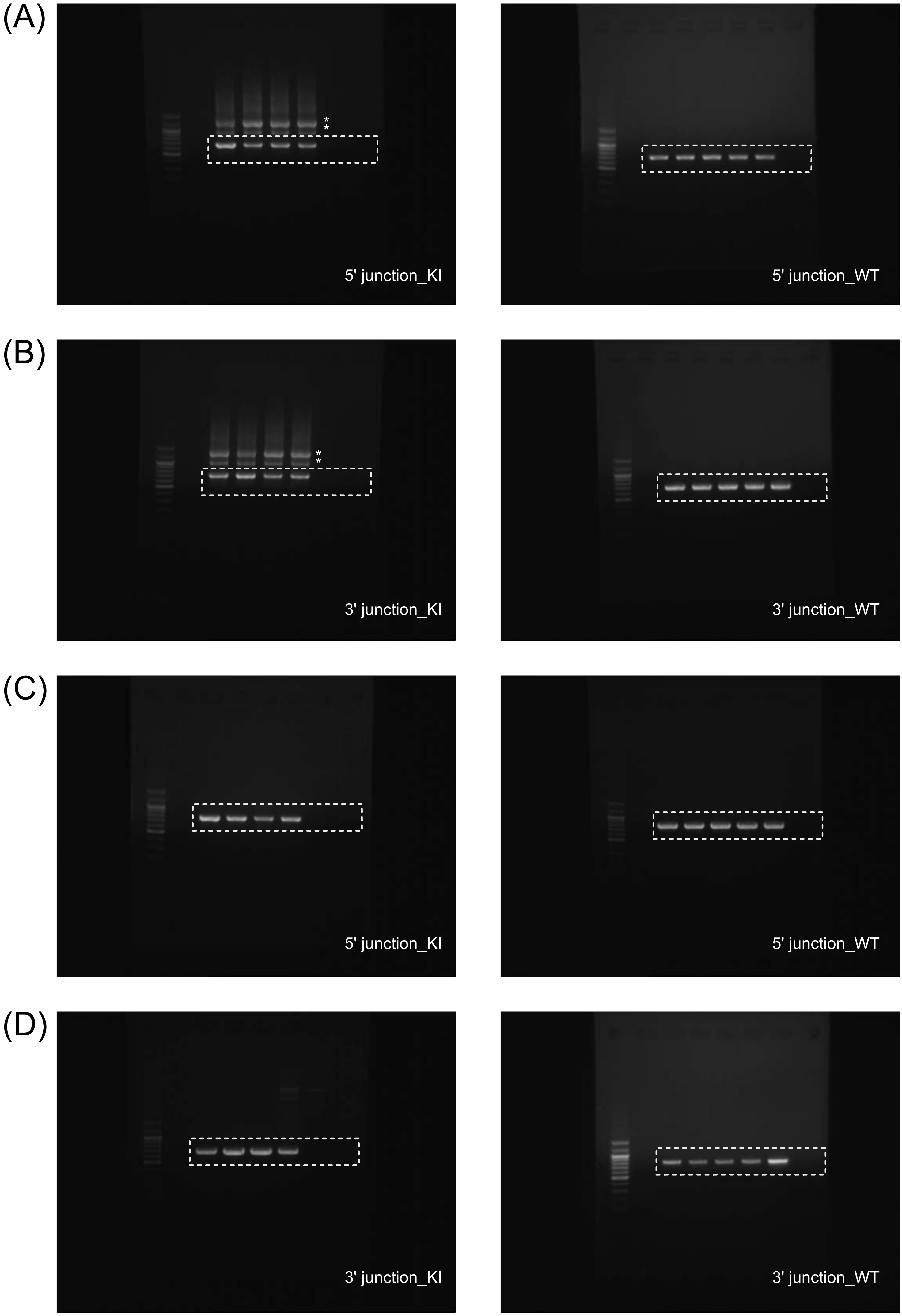
(A)
5' junction_KI
5' junction_WT
(B)
3' junction_KI
3' junction_WT
(C)
5' junction_KI
5' junction_WT
(D)
3' junction_KI
3' junction_WT

(E)

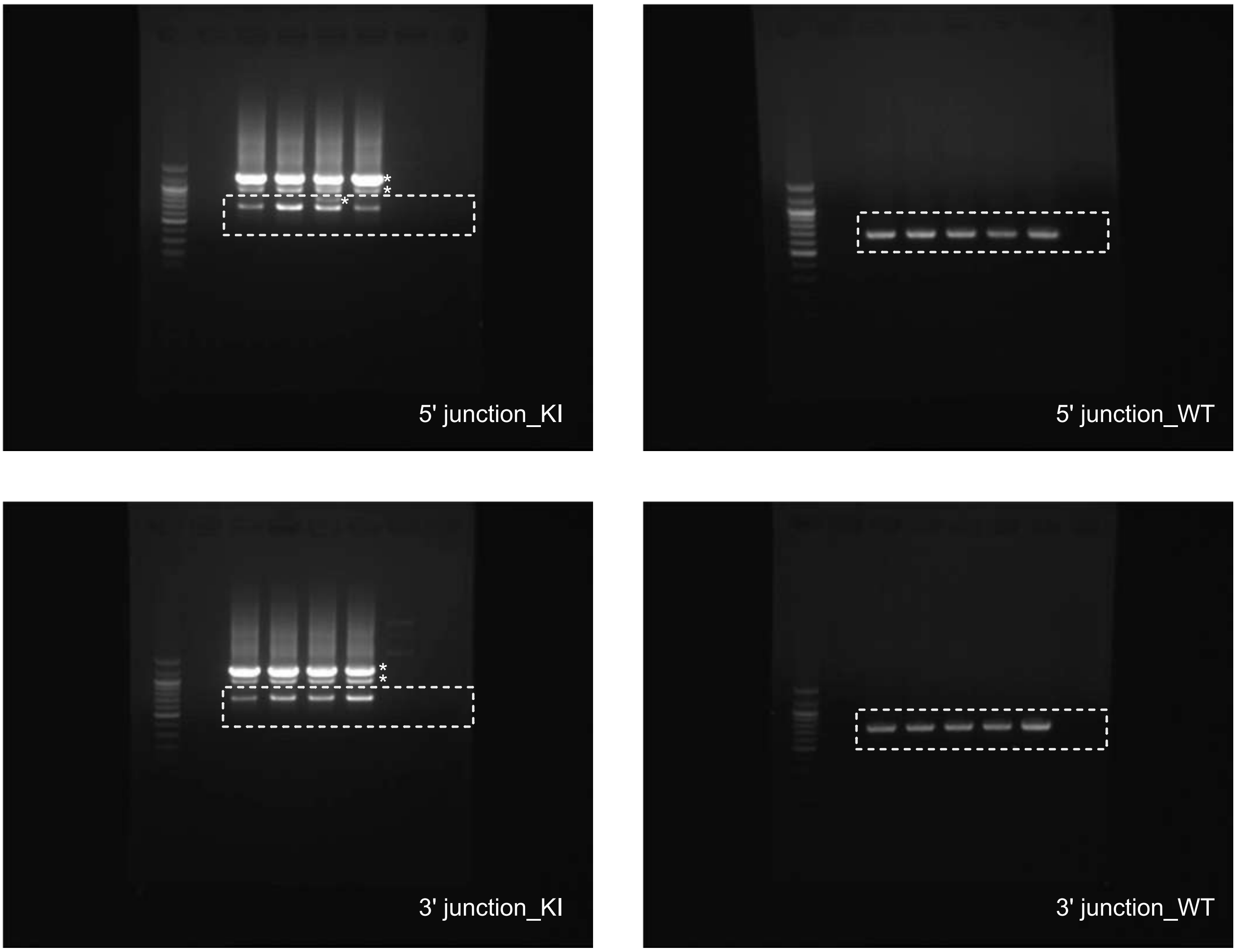

(F)

Supplementary Figure S2. Full-length, unprocessed scan for genotyping PCR gel.
Each full-length, unprocessed scan for genotyping PCR gel, (A)(B) *mCherry-LmnA*, (C)(D) *Alb-H2BmCherry*, (E)(F) *Egfr-mCherry* KI. Each dashed box area is shown in Figure 2(C), 3(C) and 4(C) respectively. Asterisk indicates non-specific amplicons. M indicates 100bp DNA ladder marker.

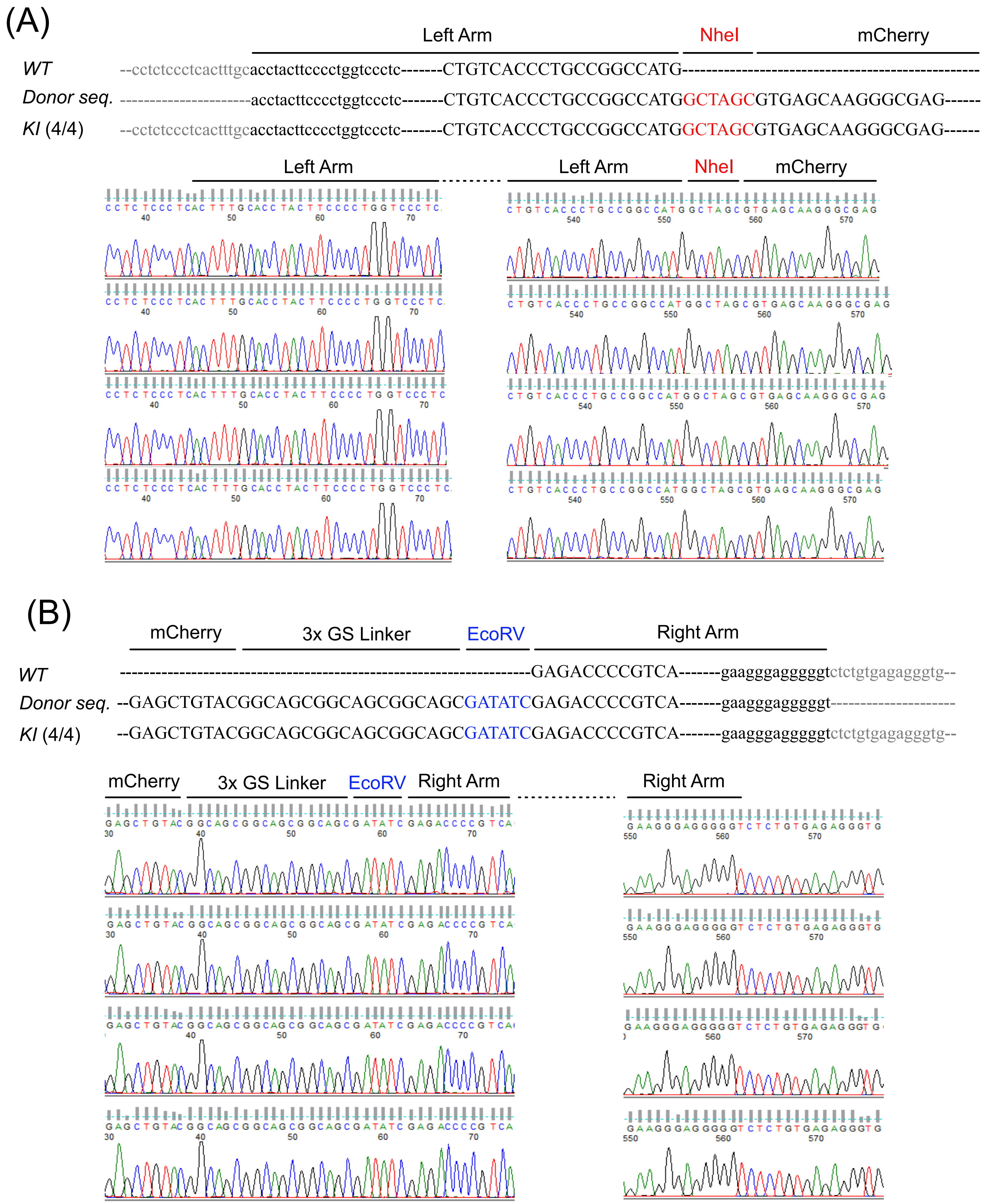

Supplementary Figure S3. Sequence analysis of *mCherry-LmnA* knock-in liver.
PCR products obtained from the 5' (A) and 3' (B) junctions of the nested PCR in Figure 2(C) were sequenced. Upper panel shows comparison of WT, donor and KI sequences. Bottom panel shows the representative result of Sanger sequencing. Grey and black letters indicate sequences inside and outside the donor cassette, respectively. Red and blue letters indicate *NheI* and *EcoRV* restriction enzyme recognition sequences, respectively. The experiment was repeated in 4 individual mice.

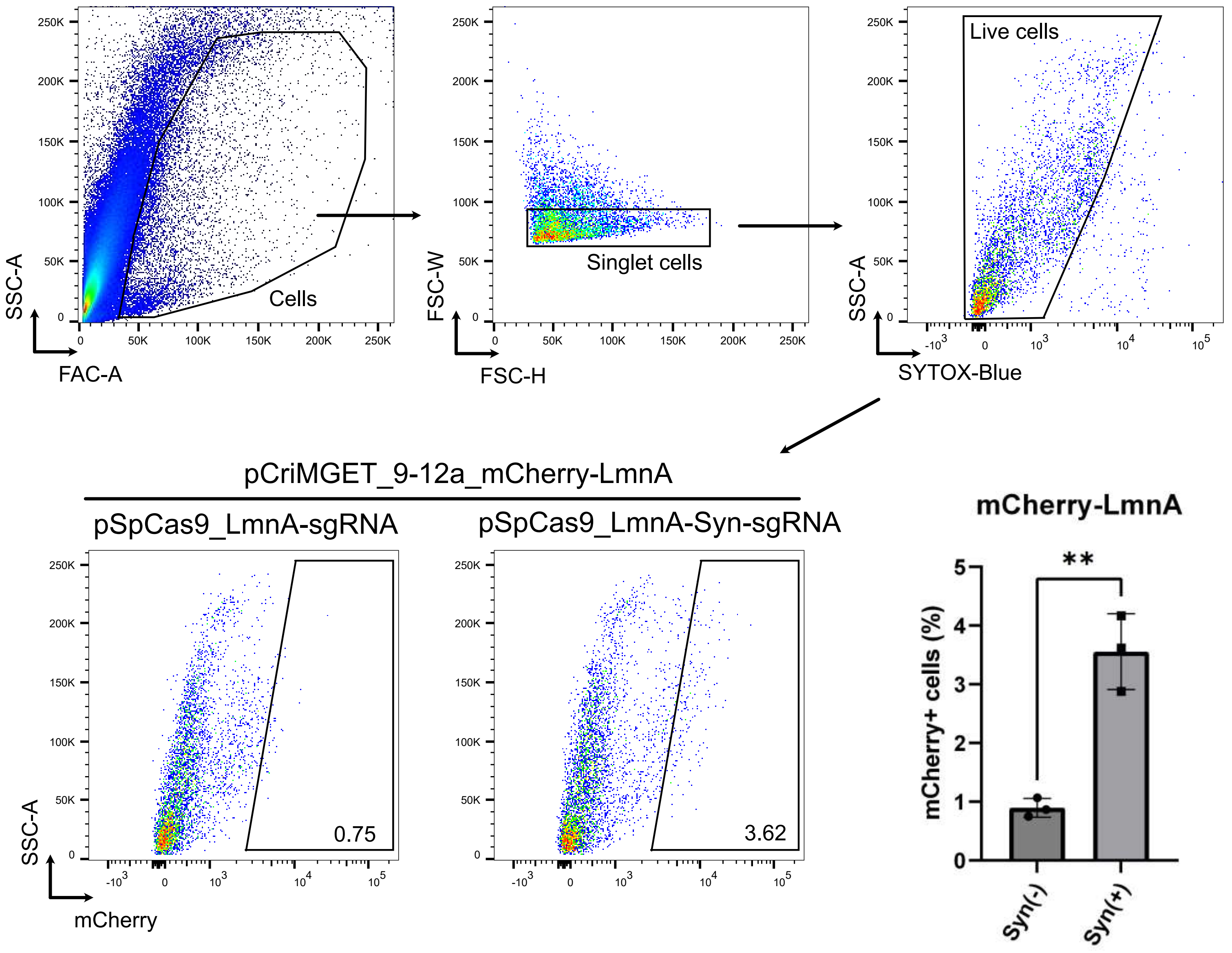

Supplementary Figure S4. Flow cytometric analysis of *mCherry-LmnA* knock-in liver.
Representative flow cytometry plots of pCriMGET_9-12a_mCherry-LmnA with pSpCas9_LmnA-sgRNA or pSpCas9_LmnA-syn-sgRNA injected liver. The total cell population was gated for forward and side scatter to identify single cell and live cell events (SYTOX Blue negative cells). Live cells were further gated and counted as mCherry positive cells. The frequency of mCherry-positive cells was calculated in 5,000 live cells. Mean ± s.d. of three individual mice. **P < 0.01, by two-tailed Student's t-test.

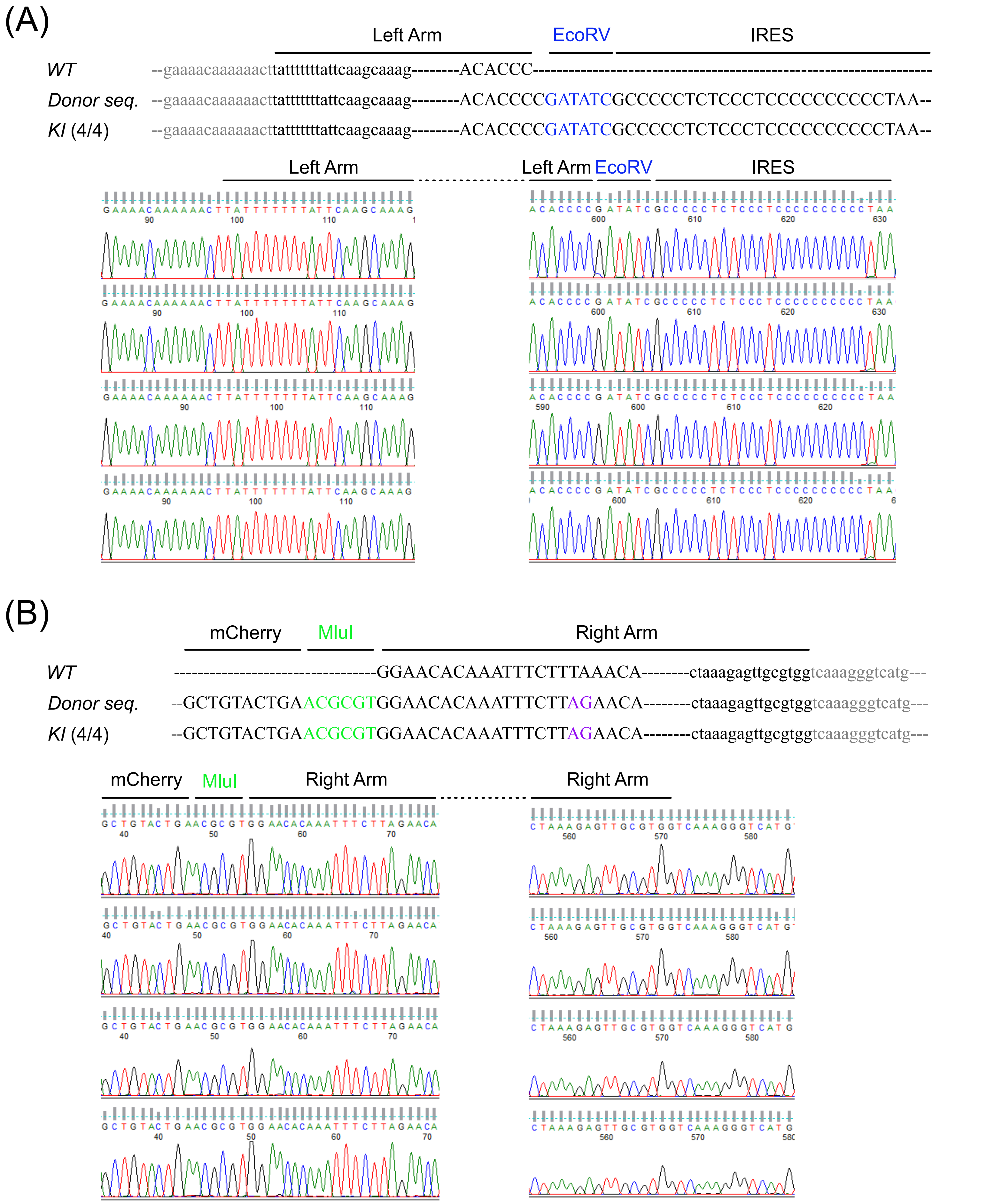

Supplementary Figure S5. Sequence analysis of *Alb-H2BmCherry* knock-in liver.
PCR products obtained from the 5' (A) and 3' (B) junctions of the nested PCR in Figure 3(C) were sequenced. Upper panel shows comparison of WT, donor and KI sequences. Bottom panel shows the representative result of Sanger sequencing. Grey and black letters indicate sequences inside and outside the donor cassette, respectively. Blue and green letters indicate *EcoRV* and *MluI* restriction enzyme recognition sequences, respectively. The experiment was repeated in 4 individual mice.

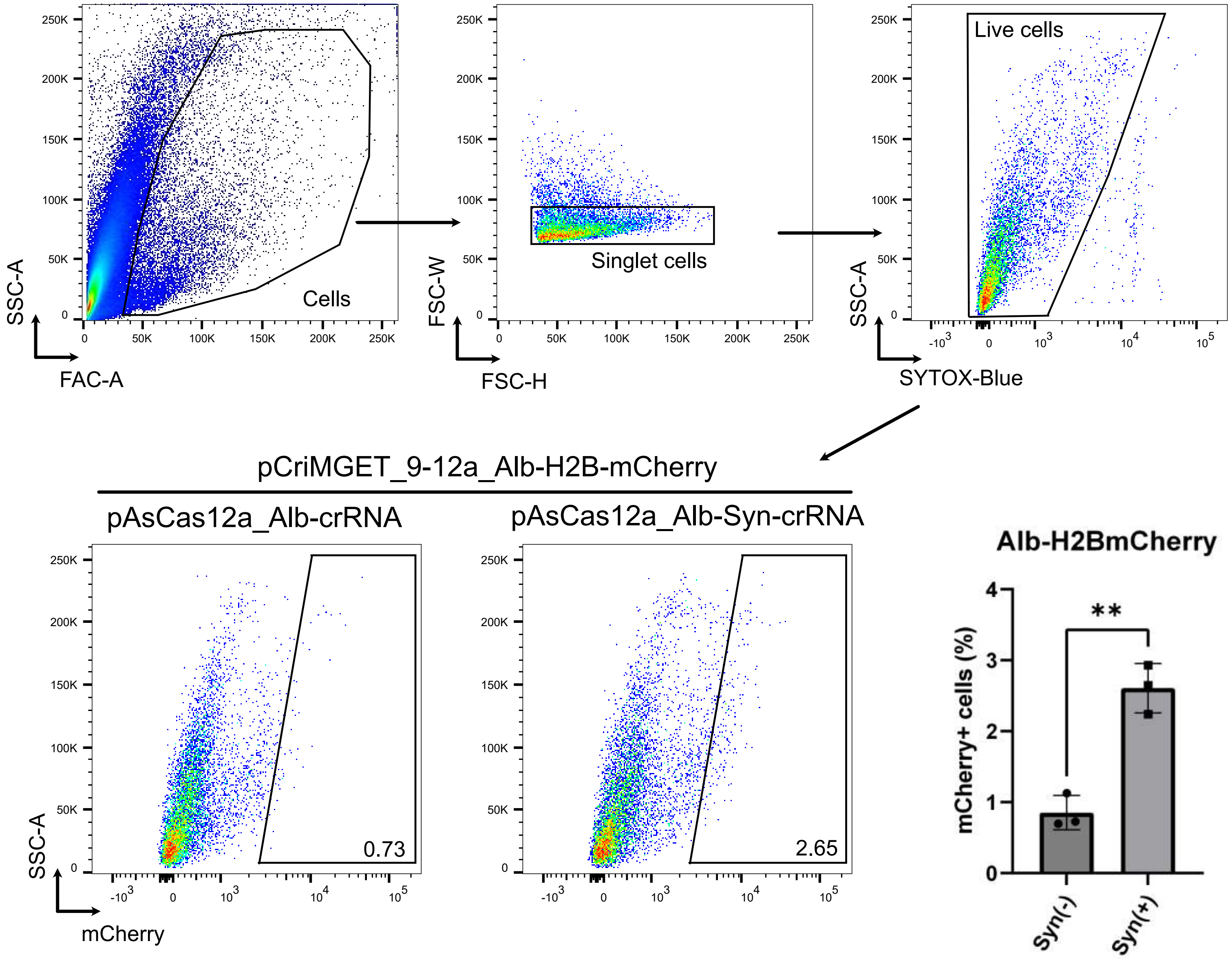

Supplementary Figure S6. Flow cytometric analysis of *Alb-H2BmCherry* knock-in liver.
Representative flow cytometry plots of pCriMGET_9-12a_Alb-H2BmCherry with pAsCas12a_Alb-crRNA or pAsCas12a_Alb-syn-sgRNA injected liver. The total cell population was gated for forward and side scatter to identify single cell and live cell events (SYTOX Blue negative cells). Live cells were further gated and counted as mCherry positive cells. The frequency of mCherry-positive cells was calculated in 5,000 live cells. Mean ± s.d. of three individual mice. **$P < 0.01$, by two-tailed Student's t-test.

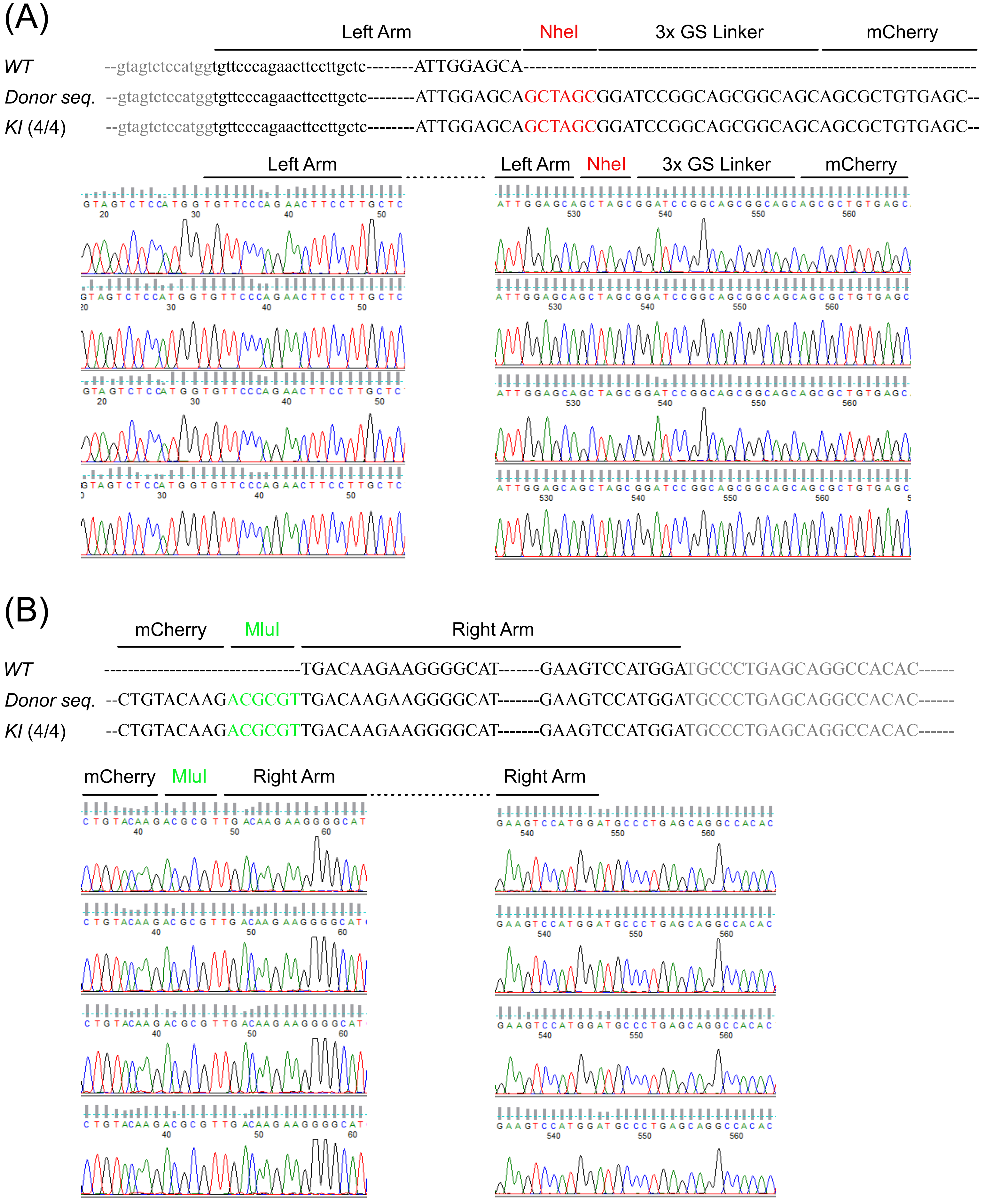

Supplementary Figure S7. Sequence analysis of *Egfr-mCherry* knock-in liver.
PCR products obtained from the 5' (A) and 3' (B) junctions of the nested PCR in Figure 4(C) were sequenced. Upper panel shows comparison of WT, donor and KI sequences. Bottom panel shows the representative result of Sanger sequencing. Grey and black letters indicate sequences inside and outside the donor cassette, respectively. Red and green letters indicate *NheI* and *MluI* restriction enzyme recognition sequences, respectively. The experiment was repeated in 4 individual mice.

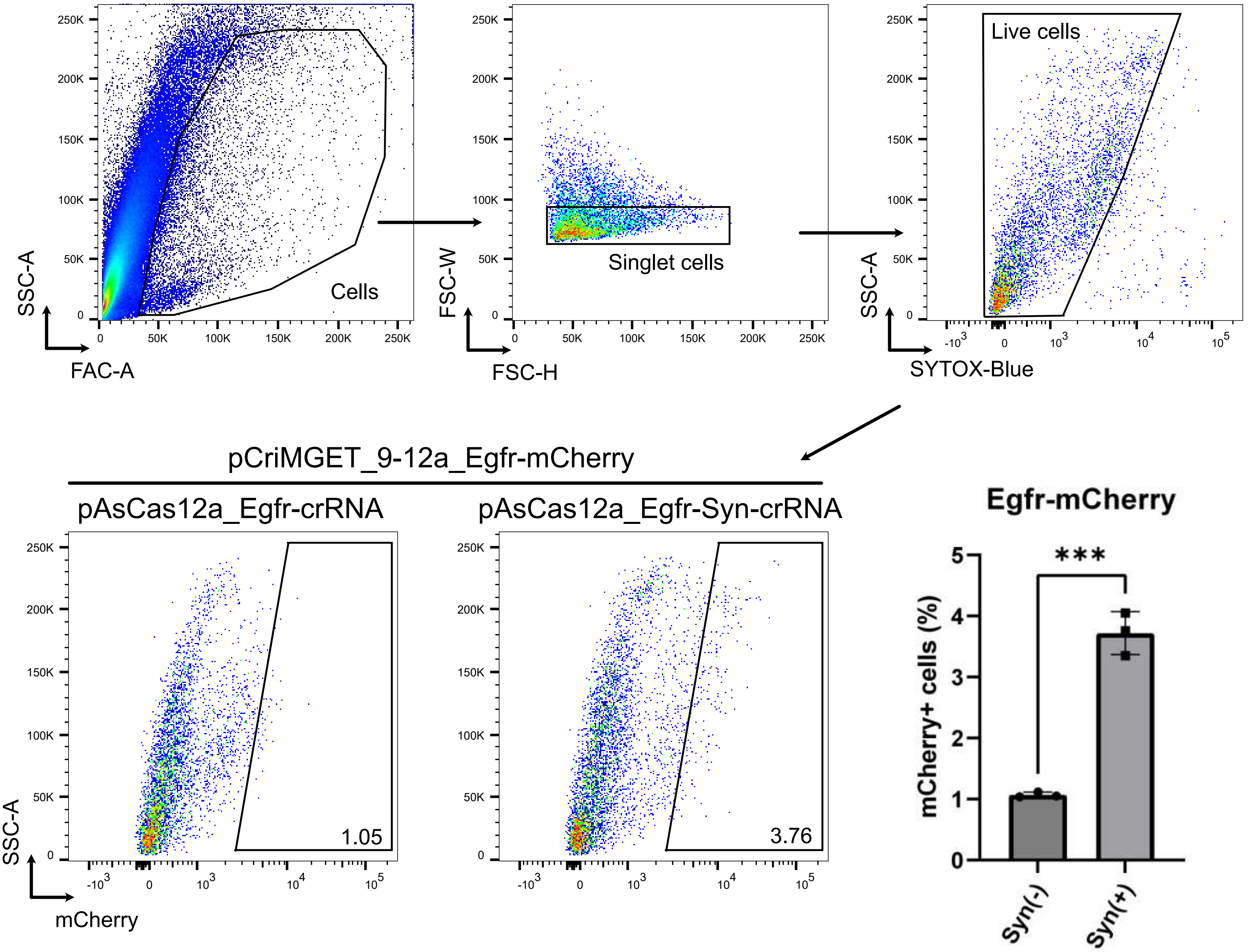

Supplementary Figure S8. Flow cytometric analysis of *Egfr-mCherry* knock-in liver.
Representative flow cytometry plots of pCriMGET_9-12a_Egfr-mCherry with pAsCas12a_Egfr-crRNA or pAsCas12a_Egfr-syn-sgRNA injected liver. The total cell population was gated for forward and side scatter to identify single cell and live cell events (SYTOX Blue negative cells). Live cells were further gated and counted as mCherry positive cells. The frequency of mCherry-positive cells was calculated in 5,000 live cells. Mean ± s.d. of three individual mice. **P < 0.01, by two-tailed Student's t-test.

Primer name	sequence (5' to 3')
* mCherry-LmnA_GT001	TCACTGATGGGCAGCAGCTTCAG
mCherry-LmnA_GT002	TGCAGGTCCTCCTTCTCCTGCAG
mCherry-LmnA_GT003	AAGCGCATGAACTCCTTGATGATG
mCherry-LmnA_GT004	AACACAGCCATTCCTTTGCCATTC
mCherry-LmnA_GT005	CCTTGGTCACCTTCAGCTTGGC
mCherry-LmnA_GT006	CCTGAGCAGCCTCTGTCCTTCTG
mCherry-LmnA_GT007	GGCCCTGTCACCAACGCTACTTG
* mCherry-LmnA_GT008	CACCATCGTGGAACAGTACGAACG
mCherry-LmnA_GT009	GAGTTCCAAGACCTGGTAGAGCCTGTTC
mCherry-LmnA_GT010	CAAGACCACCTACAAGGCCAAGAAGC
* Alb-H2BmCherry_GT001	AGCCAACTGCACACAGCTATGTTGATC
Alb-H2BmCherry_GT002	AGGAAAGGCAGGGATTCCTCTGAGC
Alb-H2BmCherry_GT003	TGCCAAAAGACGGCAATATGGTG
Alb-H2BmCherry_GT004	GTGAATCCATTTCAACAGAAGATACAACGG
Alb-H2BmCherry_GT005	ACCTTGCATTCCTTTGGCGAGAG
Alb-H2BmCherry_GT006	TAGGTCATGTGTAGGCTGTCTACACCAGTG
Alb-H2BmCherry_GT007	GGCTCCTGGAATAGAGAGAAGAACCCAG
* Alb-H2BmCherry_GT008	GAGGACTACACCATCGTGGAACAGTACG
Alb-H2BmCherry_GT009	ACAACGTCAACATCAAGTTGGACATCA
Alb-H2BmCherry_GT010	ATGTGCACAATCTGAAGCCAGGTCTG
* Egfr-mCherry_GT001	CGCTTATGCCCATTGCTTTGAGAAGTATAG
Egfr-mCherry_GT002	CAGAAGTGGAGGTGCTGCCATAGTTG
Egfr-mCherry_GT003	CATGTGCACCTTGAAGCGCATG
Egfr-mCherry_GT004	GCTTGAAAGCAAGTGGTGAAAGTGGC
Egfr-mCherry_GT005	CCTTGGTCACCTTCAGCTTGGC
Egfr-mCherry_GT006	CCAAGGAAACCAAGCCAAATGGC
Egfr-mCherry_GT007	CCGTCATTCCTCTTCTCCCAGCC
* Egfr-mCherry_GT008	ACCATCGTGGAACAGTACGAACGC
Egfr-mCherry_GT009	GACGCTGAGGTCAAGACCACCTACAAG
Egfr-mCherry_GT010	AACTAGCGCGTGTTAGGTGCCAGG

Supplementary table S1. List of primers for genotyping and sanger sequencing.
Asterisk primers (GT001 and GT008 for each gene) were also used for Sanger sequencing analysis.
